# Supplementary figures and images for: Inactivation of the mitochondrial protease Afg3l2 results in severely diminished respiratory chain activity and widespread defects in mitochondrial gene expression
Source: PLoS Genet. 2020 Oct 19;16(10):e1009118. doi: 10.1371/journal.pgen.1009118 (PMC7595625; doi:10.1371/journal.pgen.1009118)

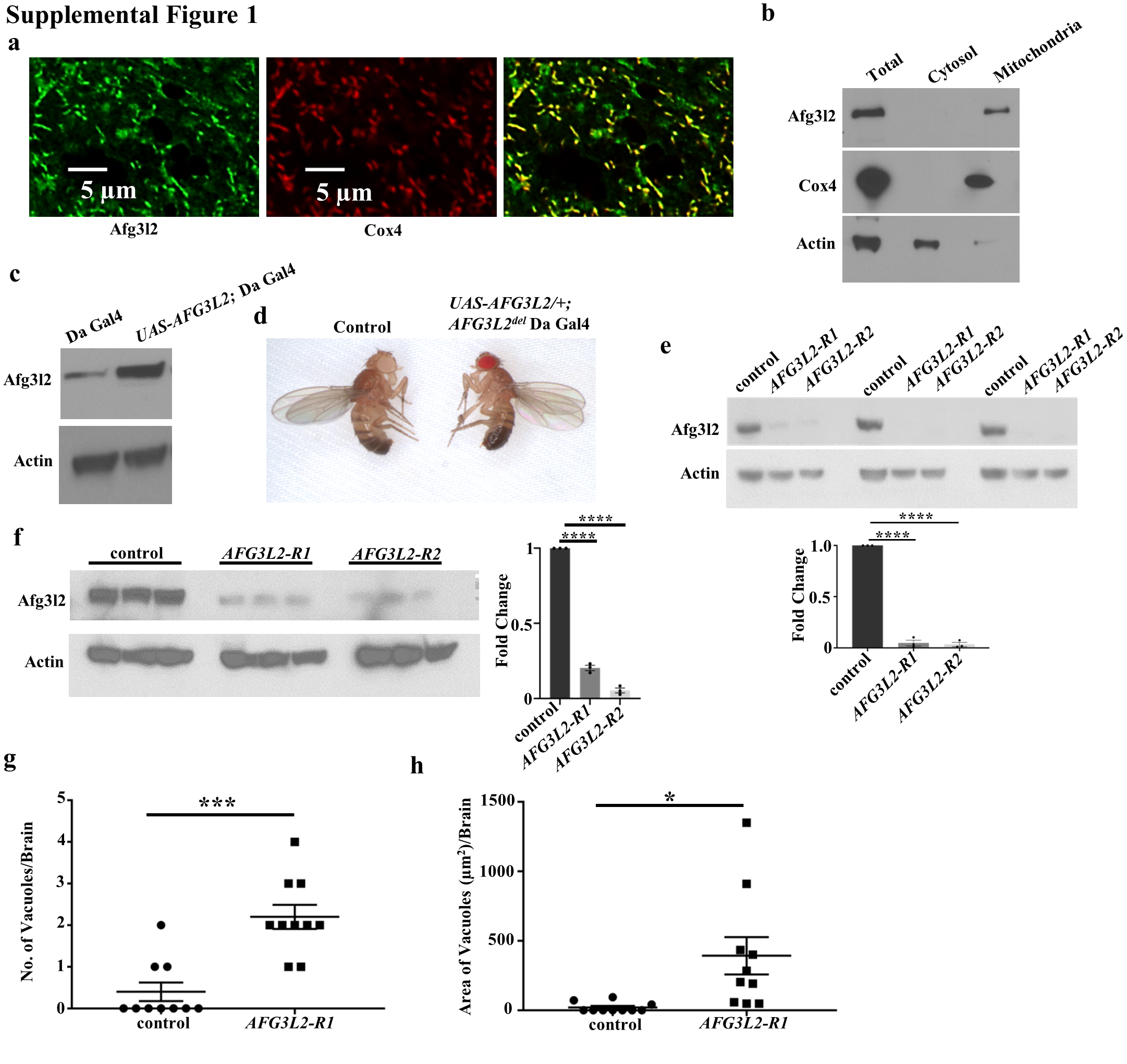

Supplement: S1 Fig — a. Confocal image of salivary glands from third instar larvae using antisera against Afg3l2 (left panel) and Cox4 (middle panel) and a merged image (right panel) showing the degree of Afg3l2 colocalization with Cox4. The scale bar is 5 μm. b. Western blot analysis of mitochondrial and cytosolic fractions from adult flies using antisera against Afg3l2, Cox4, and Actin. c. Western blot analysis of whole fly extracts from control flies and flies bearing a UAS-AFG3L2 transgene and the da-Gal4 driver. d. Image showing a control fly and a viable adult AFG3L2del homozygote rescued by ectopic expression of Afg3l2 driven by the da-Gal4 driver (AFG3L2del da-Gal4 > UAS-AFG3L2).e. Western blot analysis of protein lysates prepared from controls (UAS-LUCIFERASE RNAi/da-Gal4) and AFG3L2-deficient (UAS-AFG3L2-R1 RNAi; da-Gal4 and UAS-AFG3L2-R2 RNAi/da-Gal4) pupae 9 days following egg hatching. f. Western blot analysis of a head protein extract from 1-day old adult controls (elav-Gal4; UAS-LUCIFERASE RNAi) and age-matched flies expressing RNAi constructs targeting Afg3l2 using the neuron-specific elav-Gal4 driver (elav-Gal4; UAS-AFG3L2-R1 RNAi and elav-Gal4; UAS-AFG3L2-R2 RNAi). The band intensities were normalized to actin (N = 3, ****p < 0.0001 by one-way ANOVA Tukey's test for multiple comparison). The number (g) and area (h) of brain vacuoles in 1-day old adult fly heads of controls (elav-Gal4; UAS-LUCIFERASE RNAi) and flies expressing the weaker RNAi line targeting AFG3L2 (elav-Gal4; UAS-AFG3L2-R1 RNAi) throughout the nervous system (N = 6 fly heads, *p < 0.05, ***p <0.0005 by Student’s t-test). (TIF) [file pgen.1009118.s001.TIF]

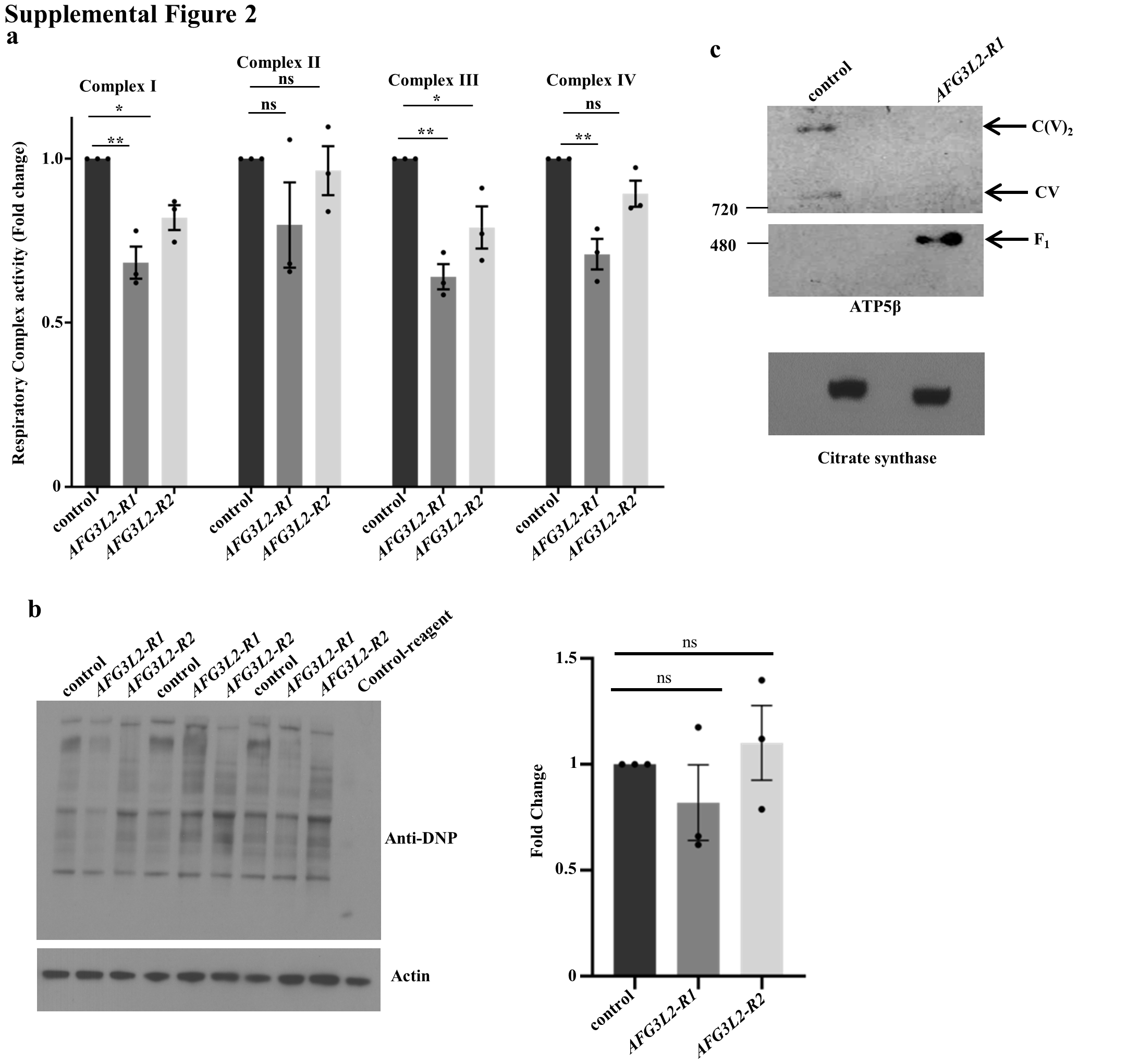

Supplement: S2 Fig — a. RC activity was analyzed using mitochondria isolated from control (UAS-LUCIFERASE RNAi/da-Gal4) and AFG3L2-deficient (UAS-AFG3L2-R1 RNAi; da-Gal4 and UAS-AFG3L2-R2 RNAi/da-Gal4) 3rd instar larvae (N = 3 independent biological replicates, *p < 0.05, **p < 0.005 by one-way ANOVA Tukey's test for multiple comparisons). b. Protein extracts from control (UAS-LUCIFERASE RNAi/da-Gal4) and AFG3L2-deficient (UAS-AFG3L2-R1 RNAi; da-Gal4 and UAS-AFG3L2-R2 RNAi/da-Gal4) pupae were subjected to western blot analysis using an antiserum to Dinitrophenyl to detect protein carbonylation. The rightmost lane in the blot represents a control sample without reagent to detect non-specific background signal from the Dinitrophenyl antiserum. The band intensities were normalized to actin. Significance was determined using one-way ANOVA Tukey's test for multiple comparisons. c. Mitochondrial protein extracts from control (UAS-LUCIFERASE RNAi/da-Gal4) and AFG3L2-deficient (UAS-AFG3L2-R1 RNAi; da-Gal4 and UAS-AFG3L2-R2 RNAi/da-Gal4) pupae were subjected to BN-PAGE analysis followed by immunoblotting using ATP5β antibody. A sub-complex containing the F1 subunit of ATP synthase was detected in AFG3L2-deficient animals, but not in controls. Citrate synthase was used as a loading control. (TIF) [file pgen.1009118.s002.TIF]

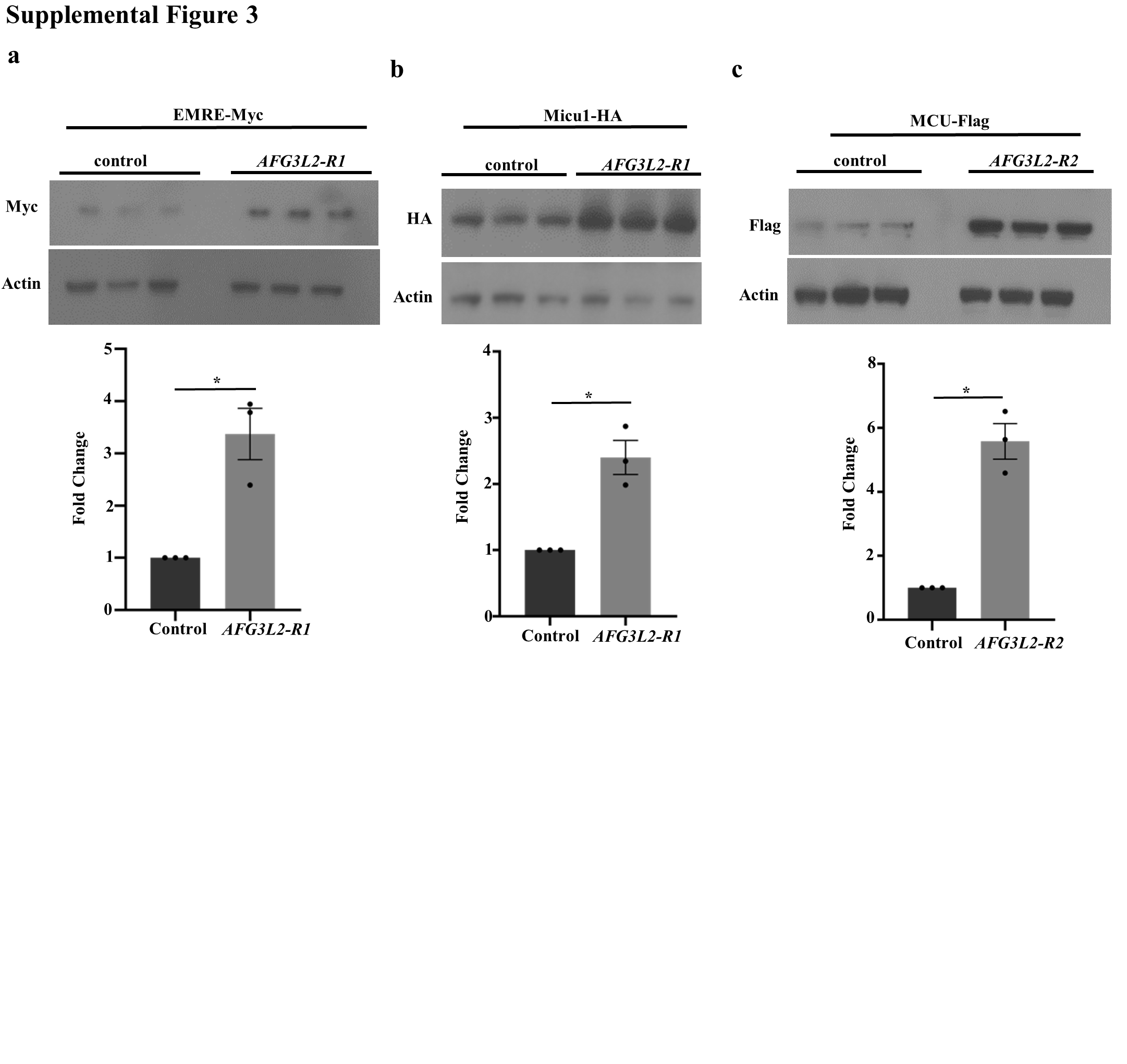

Supplement: S3 Fig — a. Cell lysates prepared from control (UAS-LEXA RNAi; UAS-EMRE-MYC/da-Gal4) and AFG3L2-deficient (UAS-AFG3L2-R1 RNAi; UAS-EMRE-MYC/da-Gal4) pupae expressing a Myc-tagged form of EMRE were subjected to western blot analysis using an antiserum against Myc. b. Cell lysates prepared from control (UAS-LEXA RNAi; UAS-MICU1-HA/da-Gal4) and AFG3L2-deficient (UAS-AFG3L2-R1 RNAi; UAS-MICU1-HA/da-Gal4) pupae expressing an HA-tagged form of Micu1 were subjected to western blot analysis using an antiserum against HA. c. Cell lysates prepared from control (UAS-MCU-FLAG; UAS-LUCIFERRASE RNAi/da-Gal4) and AFG3L2-deficient (UAS-MCU-FLAG; UAS-AFG3L2-R2 RNAi/da-Gal4) pupae expressing a Flag-tagged form of Mcu were subjected to western blot analysis using an antiserum against Flag. Band intensities were normalized against actin (N = 3 independent biological replicates, *p < 0.05 by Student’s t-test). (TIF) [file pgen.1009118.s003.TIF]

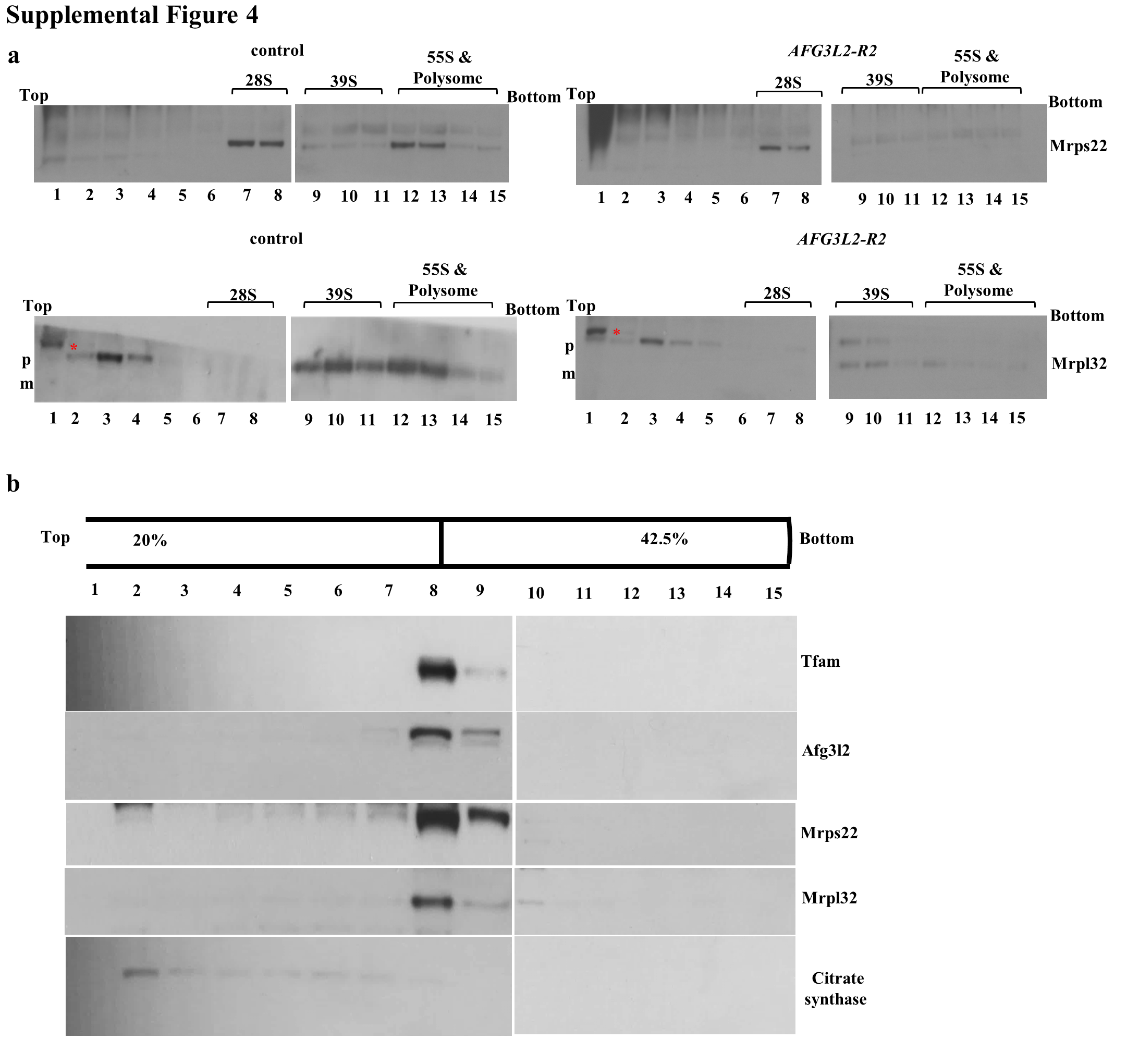

Supplement: S4 Fig — a. Mitochondrial protein fractions from controls (UAS-LUCIFERASE RNAi/da-Gal4) and Afg3l2- deficient (UAS-AFG3L2-R2 RNAi/da-Gal4) pupae were subjected to sucrose density gradient sedimentation. Individual fractions from the gradient were subjected to western blot analysis using antisera against Mrps22 and Mrpl32. Red asterisks denote a non-specific band. Data shown represent independent biological replicates. b. A mitochondrial protein lysate from control (UAS-LUCIFERASE RNAi/da-Gal4) pupae was subjected to iodixanol density gradient analysis. Fractions from the gradient were then subjected to western blot analysis using antisera against Tfam, Afg3l2, citrate synthase, Mrpl32, and Mrps22. (TIF) [file pgen.1009118.s004.TIF]

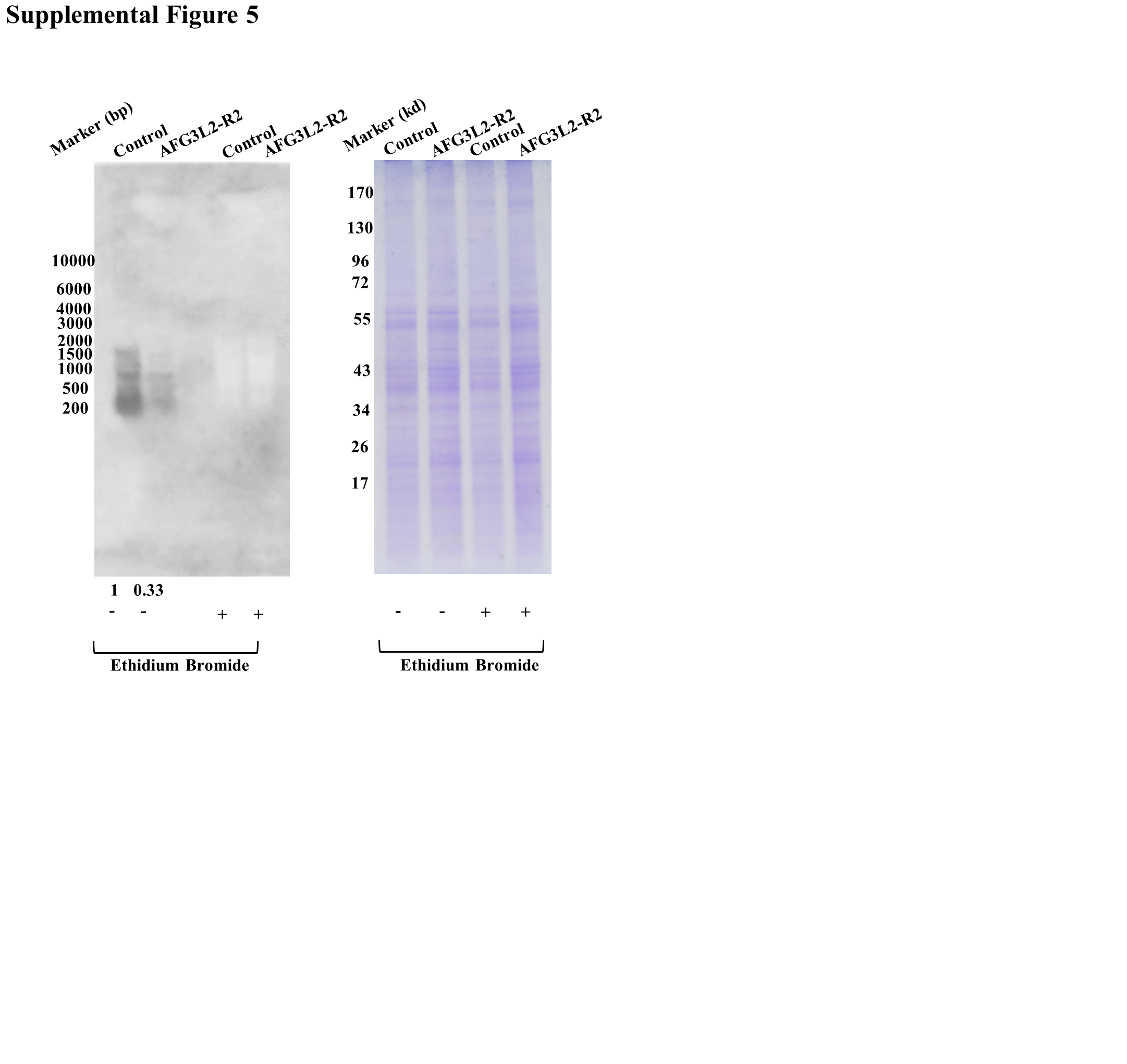

Supplement: S5 Fig — In organello transcription assay from mitochondria of control (UAS-LUCIFERASE RNAi/da-Gal4) and AFG3L2-deficient (UAS-AFG3L2-R2 RNAi/da-Gal4) pupae. Ethidium bromide, a specific inhibitor of mitochondrial transcription, was included to ensure the lack of nuclear contamination in our samples. The right panel shows a Coomassie-stained gel from the same mitochondrial preparation used to normalize protein loading (control samples defined as 1). (TIF) [file pgen.1009118.s005.TIF]

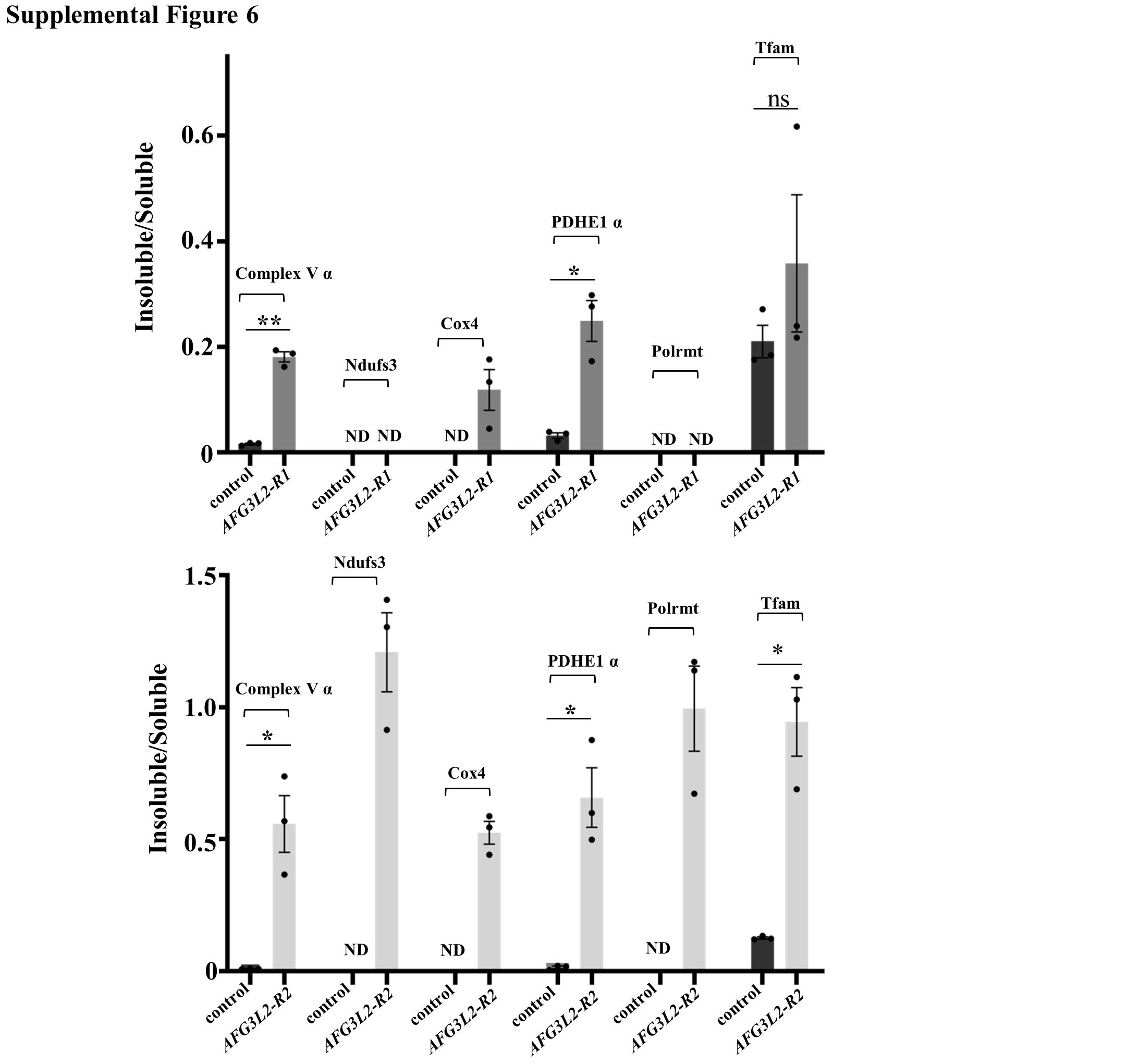

Supplement: S6 Fig — The ratio of detergent-insoluble to soluble proteins was quantified by normalizing band intensities to actin. Not determined (ND) indicates that the ratio of insoluble to soluble protein was not quantified because no insoluble material was detected (N = 3 independent biological replicates, *p < 0.05, **p < 0.005 by Student’s t-test). (TIF) [file pgen.1009118.s006.TIF]

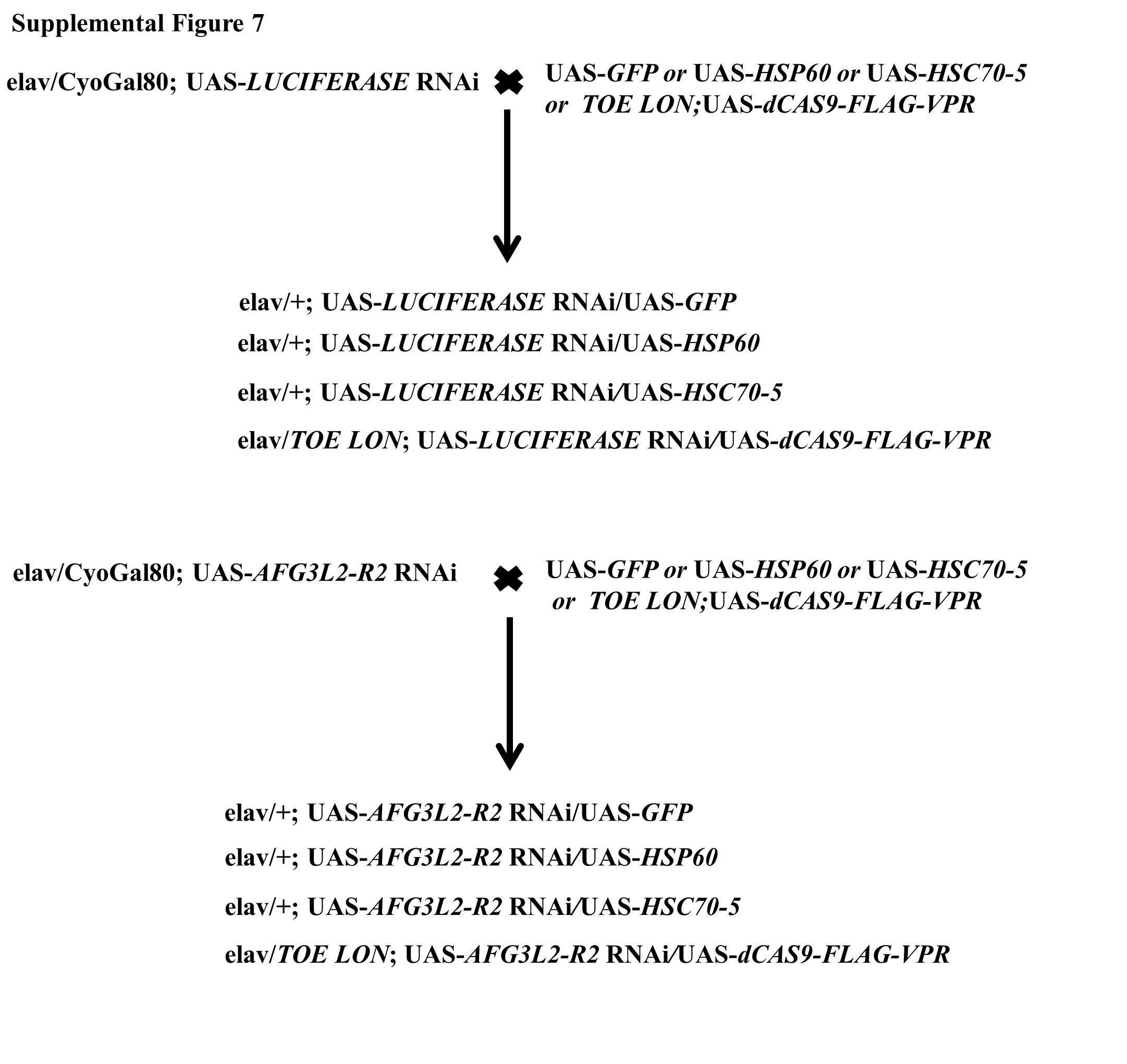

Supplement: S7 Fig — (TIF) [file pgen.1009118.s007.TIF]

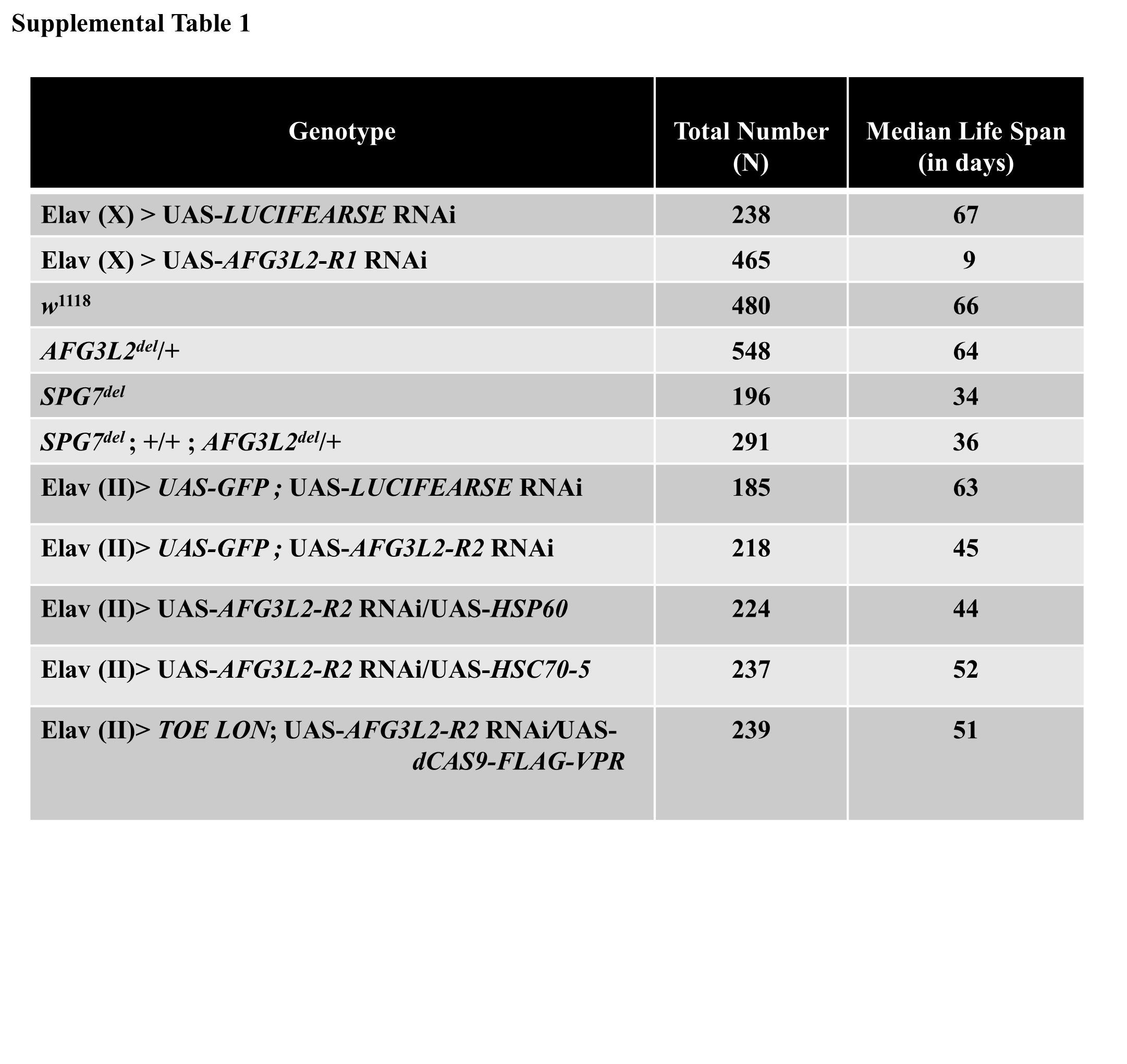

Supplement: S1 Table — Note that two different elav-Gal4 transgenes were utilized in our studies. The elav-Gal4 transgene situated on the ‘X’ chromosome (Elav (X)) was used for all initial characterization of UAS-AFG3L2-R1 and UAS-AFG3L2-R2 RNAi flies. The elav-Gal4 transgene on the 2nd chromosome (Elav (II)) was utilized for all overexpression studies. (TIF) [file pgen.1009118.s008.TIF]

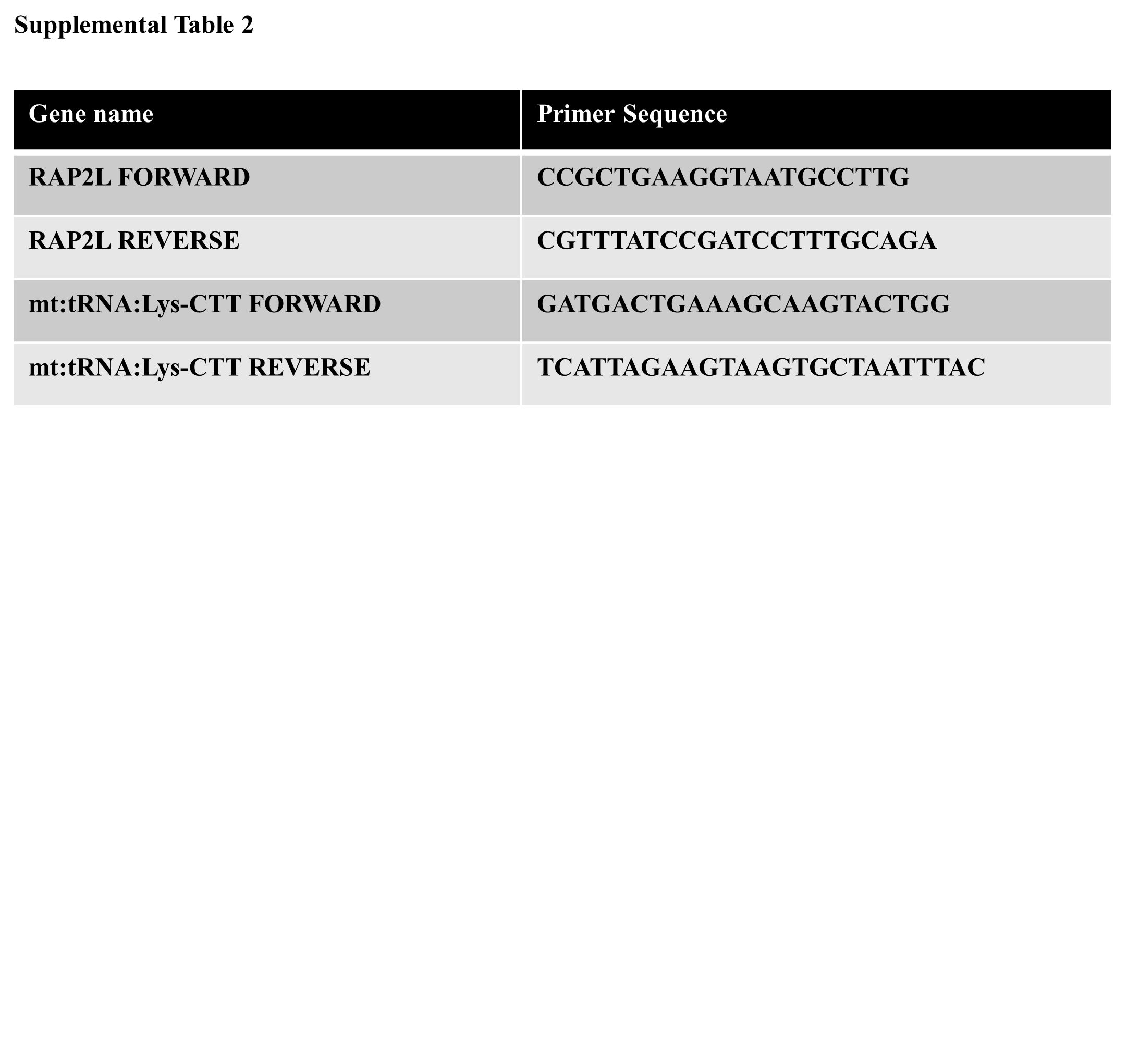

Supplement: S2 Table — (TIF) [file pgen.1009118.s009.TIF]
